# PathDIP 5: improving coverage and making enrichment analysis more biologically meaningful

**DOI:** 10.1093/nar/gkad1027

**Published:** 2023-11-22

**Authors:** Chiara Pastrello, Max Kotlyar, Mark Abovsky, Richard Lu, Igor Jurisica

**Affiliations:** Osteoarthritis Research Program, Division of Orthopedic Surgery, Schroeder Arthritis Institute, Toronto, Ontario M5T 0S8, Canada; Data Science Discovery Centre for Chronic Diseases, Krembil Research Institute, Krembil Discovery Tower, Toronto, ON M5T 0S8, Canada; Osteoarthritis Research Program, Division of Orthopedic Surgery, Schroeder Arthritis Institute, Toronto, Ontario M5T 0S8, Canada; Data Science Discovery Centre for Chronic Diseases, Krembil Research Institute, Krembil Discovery Tower, Toronto, ON M5T 0S8, Canada; Osteoarthritis Research Program, Division of Orthopedic Surgery, Schroeder Arthritis Institute, Toronto, Ontario M5T 0S8, Canada; Data Science Discovery Centre for Chronic Diseases, Krembil Research Institute, Krembil Discovery Tower, Toronto, ON M5T 0S8, Canada; Osteoarthritis Research Program, Division of Orthopedic Surgery, Schroeder Arthritis Institute, Toronto, Ontario M5T 0S8, Canada; Data Science Discovery Centre for Chronic Diseases, Krembil Research Institute, Krembil Discovery Tower, Toronto, ON M5T 0S8, Canada; Osteoarthritis Research Program, Division of Orthopedic Surgery, Schroeder Arthritis Institute, Toronto, Ontario M5T 0S8, Canada; Data Science Discovery Centre for Chronic Diseases, Krembil Research Institute, Krembil Discovery Tower, Toronto, ON M5T 0S8, Canada; Departments of Medical Biophysics and Computer Science, and Faculty of Dentistry, University of Toronto, Toronto, ON M5G 1L7, Canada; Institute of Neuroimmunology, Slovak Academy of Sciences, Bratislava, Slovakia

## Abstract

Pathway Data Integration Portal (PathDIP) is an integrated pathway database that was developed to increase functional gene annotation coverage and reduce bias in pathway enrichment analysis. PathDIP 5 provides multiple improvements to enable more interpretable analysis: users can perform enrichment analysis using all sources, separate sources or by combining specific pathway subsets; they can select the types of sources to use or the types of pathways for the analysis, reducing the number of resulting generic pathways or pathways not related to users’ research question; users can use API. All pathways have been mapped to seven representative types. The results of pathway enrichment can be summarized through knowledge-based pathway consolidation. All curated pathways were mapped to 53 pathway ontology-based categories. In addition to genes, pathDIP 5 now includes metabolites. We updated existing databases, included two new sources, PathBank and MetabolicAtlas, and removed outdated databases. We enable users to analyse their results using Drugst.One, where a drug-gene network is created using only the user's genes in a specific pathway. Interpreting the results of any analysis is now improved by multiple charts on all the results pages. PathDIP 5 is freely available at https://ophid.utoronto.ca/pathDIP.

## Graphical Abstract

**Graphical Abstract:**
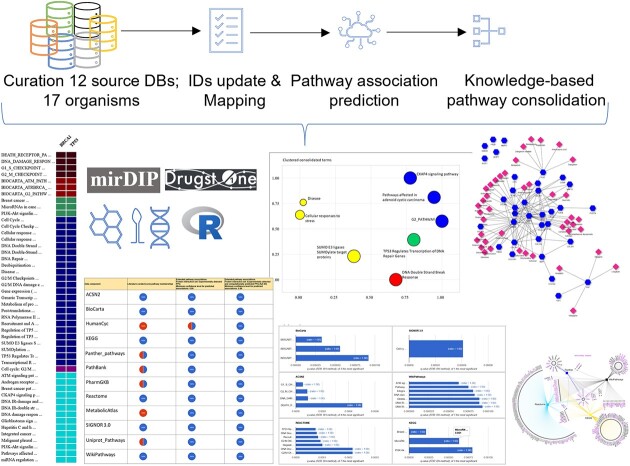

## Introduction

Pathway enrichment analysis is a powerful method to help interpret the collaborative role and molecular functions performed by a set of genes, proteins or non-coding RNAs. Increasingly, multi-omics and single-cell analyses are further enlarging the number and size of lists of genes or proteins of interest that require interpretation and characterization of the tasks performed in a cell. Because pathways are entities created by researchers to describe a phenomenon, there is large variability in the representation of each entity. This leads to poor overlap among databases (1), and the rationale behind the creation of Pathway Data Integration Portal (PathDIP) (2). Since its first release, we have increased the number of databases included and extended the gene coverage across organisms, to try to depict the most complete picture available for each pathway in multiple species. One side effect of this approach has always been the large number of human-curated pathways, which creates a challenge with multiple-testing correction, and sometimes results in lists of pathways that are too long and challenging to summarize and interpret.

Pathway consolidation, the process of unifying pathways from multiple databases, is a powerful method aimed to help reduce the number of pathways while increasing the completeness of pathway coverage and characterization. While the goal is clear, its implementation is non-trivial in an automated fashion, assuring consistency, and with an aim of biological relevance. Pathway databases create some challenges in the integration of similar pathways, as discussed in (3). We address this challenge in PathDIP 5 by providing more specific pathway enrichment filters and annotations, and knowledge-based pathway consolidation. We created an integrated ontology using KEGG and Reactome and mapped all 6535 human-curated pathways to 53 representative categories, aiming to reduce and prioritise lists of pathways and make it easier for researchers to perform function-specific validation *in vitro* or *in vivo*, creating a valuable feedback loop between experimental laboratory and computational analyses.

## Materials and methods

### Data collection, processing and membership prediction

#### Core pathways

We removed 10 source databases from pathDIP as they have not been updated in the past 10 years. Moreover, SMPDB is now part of PathBank, and NetPath is now included in WikiPathways; for this reason, the original SMPDB and NetPath were removed. We added PathBank and MetabolicAtlas as new sources and collected updated versions of the remaining 10 databases (May 2023). We excluded from the collected data all the pathways that included only one gene (*n* = 2776). Table 1 lists the details for each database, including the non-human organisms we curated from them and whether they included metabolite species.

**Table 1.** List of databases included in pathDIP 5

Source	Downloaded from	Citation	Organisms*	Metabolites
*ACSN2*	Source website	(4)	Hs	No
*BioCarta*	MSigDB	(5)	Hs	No
*HumanCyc* *	PathwayCommons	(6)	Hs	No
*KEGG*	CPDB	(7)	Hs, Mm, Sc	Yes
*MetabolicAtlas*	Source website	(8)	Hs, Ce, Dm, Mm, Rn, Sc	Yes
*Panther*	Source website	(9)	Hs, Bt, Ce, Cf, Dm, Ec, Gg, Mm, Rn, Sc, Ss	No
*PathBank*	Source website	(10)	Hs, Bt, Ce, Dm, Mm, Rn, Sc	Yes
*PharmGKB*	Source website	(11)	Hs	Yes
*Reactome*	Source website	(12)	Hs, Bt, Ce, Cf, Dm, Gg, Mm, Rn, Sc, Ss	Yes
*SIGNOR* 3.0	Source website	(13)	Hs	Yes
*UniPro t.P athways*	Source website	(14)	Hs, Bt, Ce, Cf, Cp, Dm, Ec, Gg, Mm, Oa, Oc, Rn, Sc, Ss	No
*WikiPathways*	Source website	(15)	Hs, Bt, Ce, Cf, Dm, Ec, Gg, Mm, Rn, Sc, Ss	No

*Hs = Homo sapiens, Bt = Bos taurus, Ce = Caenorhabditis elegans, Cf = Canis lupus familiaris, Cp = Cavia porcellus, Dm = Drosophila melanogaster, Ec = Equus caballus, Gg = Gallus gallus, Mm = Mus musculus, Oa = Ovis aries, Oc = Oryctolagus cuniculus, Rn = Rattus Norvegicus, Sc = Saccharomyces cerevisiae, Ss = Sus scrofa

#### Protein IDs, orthologs and interactions

Mappings between UniProt IDs, NCBI Gene IDs, gene symbols and protein names were obtained from HGNC (16) (2023-08-30) and genekitr (17) version 1.2.2. We have switched Primary IDs from NCBI Gene to UniProt, as in Reactome and IID (18). One-to-one orthologs were downloaded from Ensembl (19) release 103. Physical protein–protein interactions (PPIs) were downloaded from IID (18) version 2021–05.

#### Predictions based on orthology

For each non-human organism, we replaced members of *core* human pathways with their orthologs and kept only pathways with at least three ortholog members (i.e. we did not consider a single protein or a single interaction (two proteins) as a pathway).

#### Predictions based on physical network connectivity

As in the previous versions, we predicted statistically significant protein-pathway associations for each species in pathDIP 5, using species-specific PPIs from IID. Supplementary Table 1 shows the databases integrated in IID and used for the prediction. Due to the continuously increasing size and density of the interactome and the number of curated pathways, we modified the method used since version 1 to increase reliability. We predicted an association between a protein, *prot_i_*, and a pathway, *path_j_*, if *prot_i_* had a significant number of interaction partners that were members of *path_j_*, according to core pathway data. Significance was calculated in two steps. First, the probability of protein, *prot_i_*, having at least *k* interaction partners from pathway, *path_j_*, was calculated using a hypergeometric distribution as follows:


\begin{equation*}\Pr \left( {X \ge k} \right) = \ \mathop \sum \limits_{m = k}^{{\mathrm{min}}\ \left( {n,M} \right)} \frac{{\left( {\begin{array}{@{}*{1}{c}@{}} M\\ m \end{array}} \right)\left( {\begin{array}{@{}*{1}{c}@{}} {N - M}\\ {n - m} \end{array}} \right)}}{{\left( {\begin{array}{@{}*{1}{c}@{}} N\\ n \end{array}} \right)}}\end{equation*}

where *N*= number of proteins in the PPI network, *M* = number of proteins in the PPI network that are core *path_j_* members, *n* = total number of *prot_i_* interaction partners, *m* = number of *prot_i_* interaction partners that are core *path_j_* members. Such probabilities were calculated for all core pathways involving *prot_j_* interaction partners. Second, these probabilities were adjusted for multiple testing using the Benjamini–Hochberg method (20). Pathways with adjusted probabilities <0.01 were kept as predicted pathways associations of *prot_i_*. This prediction method differs from previous pathDIP versions, where *N* was the number of network proteins with pathway annotations and *n* was the number of interaction partners with pathway annotations. The new method provided pathway annotations for 355 proteins that previously had no curated or predicted pathway annotations.

For human, we provide predicted protein-pathway associations using *core* pathways and two sets of PPIs: (i) experimentally detected PPIs and (ii) the full set of human PPIs available in IID (i.e. the combination of experimentally detected and computationally predicted physical protein interactions). For non-human species, we used only one set of PPIs, i.e. the full set of species-specific PPIs, to predict strong physical associations between each protein and each pathway in *core* (if available) or *ortholog* pathway sets.

#### Pathway types and categories

Each pathway in PathDIP 5 was mapped to one of seven pathway types and one of fifty-three pathway categories (Supplementary Table 2). Pathway types are broad topics (e.g. metabolism, disease) that can be selected prior to running enrichment analysis, to increase the relevance and statistical significance of enrichment results. Pathway categories are smaller groups of pathways sharing a similar function (e.g. carbohydrate metabolism), disease type (e.g. immune diseases), or other properties. PathDIP 5 uses categories to provide a consolidated view of enrichment results: while full enrichment results may include several thousand pathways, a consolidated view displays up to 53 pathways, each one being the most enriched pathway in its category.

Pathway types and categories are largely based on the ontology of the KEGG pathway database (21). Several new categories were added (e.g. Cellular response to stimuli, Muscular and bone system) to accommodate certain pathways not present in KEGG.

Pathways were mapped to types and categories using pathway ontologies, regular expression rules, and manual curation. Pathways from Reactome (22) and WikiPathways (23) were mapped using Reactome ontology and Pathway Ontology (24), respectively. In this approach, high-level terms in an ontology were manually assigned to categories (e.g. Reactome term ‘Metabolism of carbohydrates’ was assigned to category ‘Carbohydrate metabolism’) and then a term's descendent pathways were mapped to the term's category. Pathways from sources without ontologies were mapped either with regular expressions, if pathway names from the same category shared common patterns, or through manual curation.

#### Pathway enrichment analysis

Pathway enrichment p-values are calculated as in (13), using Fisher's Exact test. Multiple testing correction *q*-values are calculated by two methods: Bonferroni and False Discovery Rate (Benjamini–Hochberg) (8). Enrichment of a user-defined protein (gene) list, *U*-list, for a given pathway, *Pw*, is calculated as follows:


\begin{equation*}{\mathrm{p \hbox{-} value}}\left( {U{{ \hbox{-} }}list,\ Pw} \right) = \ \mathop \sum \limits_{{n}_{\underset{\raise0.3em\hbox{$\smash{\scriptscriptstyle-}$}}{u} } = {N}_{\underset{\raise0.3em\hbox{$\smash{\scriptscriptstyle-}$}}{u} }}^{{\mathrm{min}}\ \left( {{N}_U,{N}_{Pw}} \right)} \frac{{\left( {\begin{array}{@{}*{1}{c}@{}} {{N}_{Pw}}\\ {{n}_{\underset{\raise0.3em\hbox{$\smash{\scriptscriptstyle-}$}}{u} }} \end{array}} \right)\left( {\begin{array}{@{}*{1}{c}@{}} {{N}_S - {N}_{Pw}}\\ {{N}_U - {n}_{\underset{\raise0.3em\hbox{$\smash{\scriptscriptstyle-}$}}{u} }} \end{array}} \right)}}{{\left( {\begin{array}{@{}*{1}{c}@{}} {{N}_S}\\ {{N}_U} \end{array}} \right)}}\end{equation*}

where *N_s_* = number of proteins in user-selected pathway databases and pathway types (background), *N_U_* = number of proteins in *U*-list that are also in the background, *N_Pw_* = number of proteins in pathway *Pw*, *N_u_* = number of proteins in *U*-list that are also in *Pw*.

### Portal description

#### Enrichment analysis improvement

Historically, pathDIP provided pathway enrichment analysis using all available (or selected) sources combined. While this provided strong results with largely adjusted *P*-values, it could also provide vast lists of pathways or be too stringent and provide no results at all. To address both challenges, a researcher can now perform the search by source across all databases, where results are calculated and presented by the individual database source. This enables researchers to familiarise themselves with different types of pathways provided by specific sources, their relevance to the question at hand, their possible biases, and to obtain smaller adjusted *P*-values for enriched pathways. Moreover, a user can choose to obtain only results with adjusted *P*-values lower than 0.01, 0.05 or 0.1. For explorative purposes, they can also choose not to filter by adjusted *P*-value and retrieve all the available results.

We also consolidated pathways inside and across databases, providing mapping for each pathway to a type and a category. This important feature can be used either before searching or after retrieving the results. For example, pathway databases like KEGG and WikiPathways include multiple disease pathways that may not be of interest to a researcher, who would previously had to either exclude such sources or retrieve a list of pathways with pathways not of interest. In this example, the researcher can now select the types of pathways for the analysis, reducing the number of resulting generic pathways or pathways not related to the research question at hand.

Finally, pathway enrichment analysis in the microRNA page can now be performed on genes targeted by all query microRNAs, and not only on all the targets of each microRNA.

#### New features

We substantially modified pathDIP to improve its integration into complex bioinformatics workflows. As mentioned in methods, in addition to genes, pathDIP 5 also covers metabolites from each source database, and we developed a dedicated metabolic pathway enrichment analysis page.

Users can integrate pathDIP with their workflow using the API, as before, and can query microRNAs directly without going through mirDIP, as in version 4; now they can also analyse their results using Drugst.One (25), a tool that integrates proteins with their interactome and annotates them with information like targeting drugs or known disease associations. For Drugst.One, the network is created per enriched pathway using only the user's genes in that pathway.

Multiple charts have been included on all the results pages. We now provide bar charts to show the q-value for the top seven pathways in each source (if available). We also provide the ratio of annotated query proteins over the pathway size. We provide lists and pie charts of identified query genes per source and per set (i.e. literature curated or predicted) so that researchers can investigate if their query genes are more well-known and annotated across databases or if they tend to be less studied or even pathway orphans.

The Gene/Pathway matrix now is available as a heatmap style chart as well, colour-coded by source.

## Results

### PathDIP content

**Figure 1.**
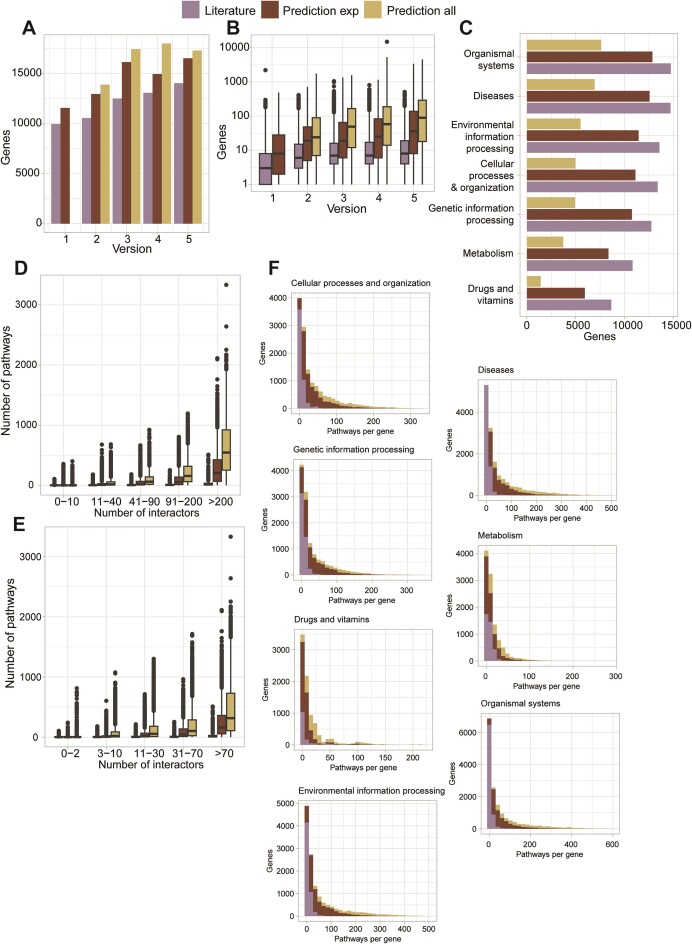
Genes and pathways distribution across pathDIP database versions. Panel **A** shows the number of genes annotated with any pathway in each version of pathDIP and for each set (curated, extended using only experimental, and extended using experimental and predicted PPIs). Panel **B** shows the distribution of the number of pathways per gene across versions and sets. For panel B, only genes present in at least two versions were considered. Panel **C** shows the number of genes present in each pathway type, for every set. Panels **D** and **E** show for each degree interval, with the degree being the number of protein interactions as identified using IID, the number of pathways the proteins are annotated with, for each set. In Panel D, protein degree was calculated using only experimental PPIs, while in Panel E it was calculated using experimental and predicted PPIs. Panel **F** shows the number of genes annotated with a certain number of pathways in each set, separated by pathway type.

PathDIP 5 now includes protein and metabolite members of 6535 human-curated pathways from 12 pathway databases. From version 1, pathDIP had significantly broader genome coverage with pathway annotation compared to any other database. Figure 1A shows how not only updated pathway curation and database integration improved the number of human-annotated proteins with consecutive versions, but also the scale of the extension of pathway orphans’ annotation. At present, 14046 human genes are annotated with curated pathways, while extended pathways provide annotation for 3236 additional genes. Moreover, as visible in Figure 1B, the number of pathways per human gene reached a plateau around version 3, while the same number has been increasing steadily across versions for predicted pathway associations. The current version also includes 5783 metabolites across species.

Interestingly, while the number of genes present in each type of pathway varies in the literature-curated sets, with the metabolic and drug-related pathways including the lowest number of genes (as expected), our predicted annotations are homogeneously and proportionally distributed for genes across categories (see Figure 1C). Figures 1D and E show that we can predict a larger number of pathways for proteins that have a higher number of protein interactions, as expected, but also that proteins with a larger number of interactions have a larger number of pathway annotations. This is very likely due to the well-known study bias, where ‘famous’ proteins (such as TP53) are studied in depth and are annotated with multiple functions, while less-known proteins lack interactions, annotations or both. Using predicted protein interactions provides a way to obtain protein interactions for less-known proteins, and to predict their pathway annotations, giving a researcher studying protein and pathway orphans the same ability to identify relevant molecular functions as a researcher studying more famous proteins. Thus, reducing bias. When considering the number of pathways per gene, we can see that most genes are annotated with a large number of pathways and that our predictions add a large number of pathways per gene only in the metabolic and drug-related pathway types (Figure 1F).

**Figure 2.**
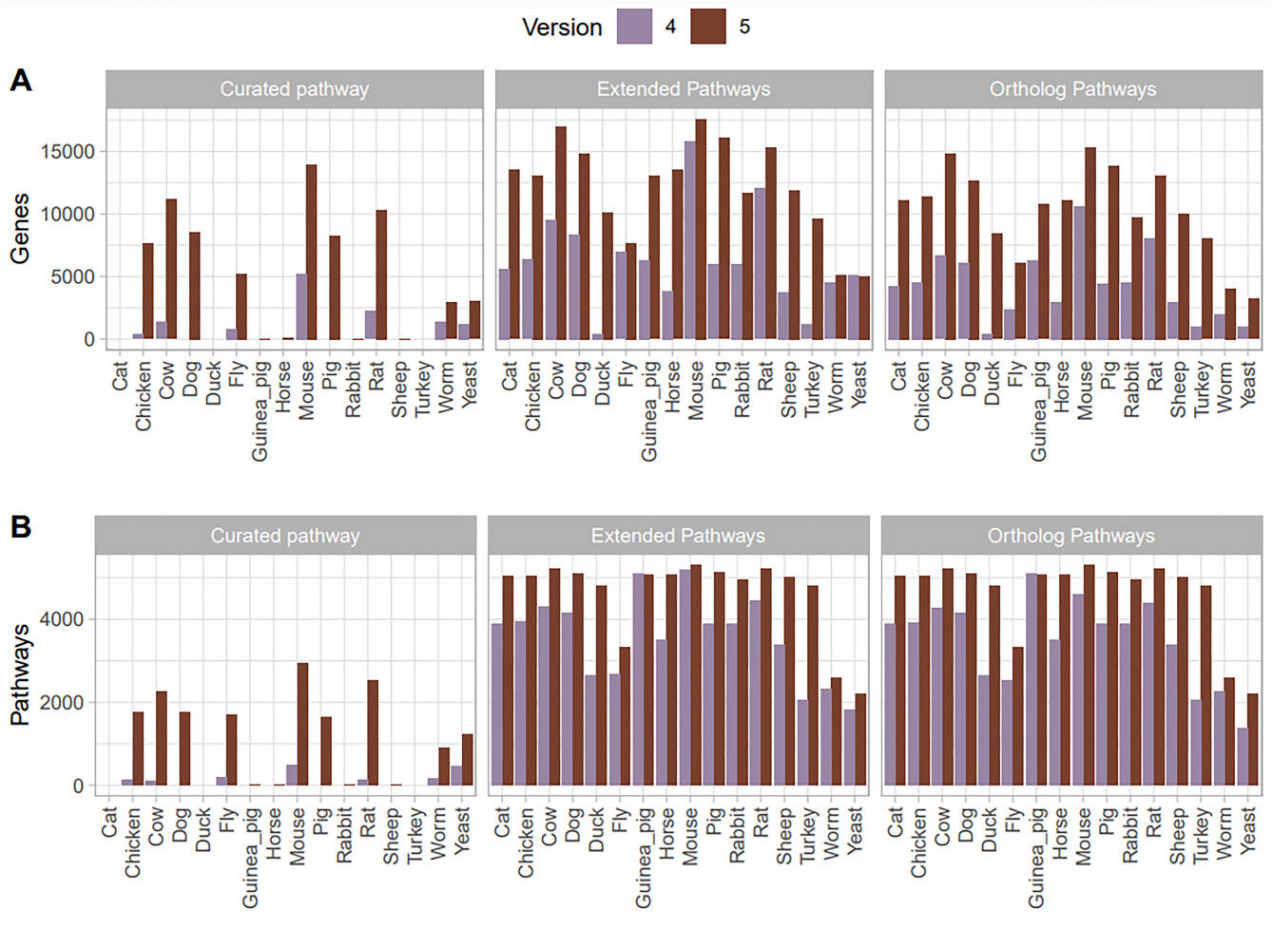
Number of genes (**A**) and pathways (**B**) present in each species (excluded human) in pathDIP 5 compared to the numbers in pathDIP 4. Numbers are shown per each set (i.e. curated or predicted).

The number of organisms other than human for which curated pathways are available is now 13, from 7 in the previous version. For the 7 in common, the number of curated pathways is larger in pathDIP 5: it varies by organism, but it goes from doubling the number (in yeast) to 20 times more pathways (in cow). Moreover, the number of pathways curated or predicted per organism increased as well (30% median across organisms), as shown in Figure 2.

### Example applications

#### Value of pathway consolidation

**Figure 3.**
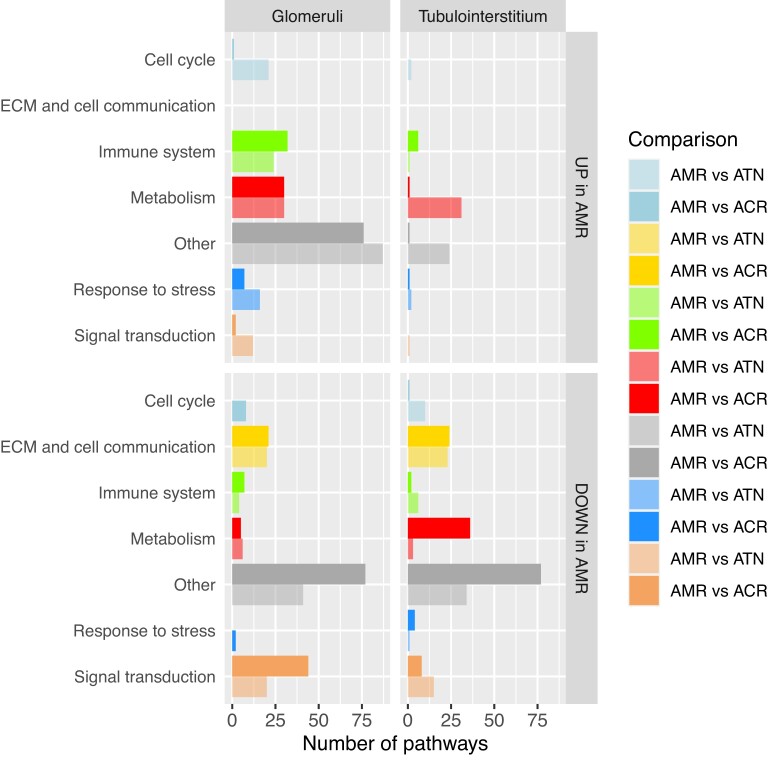
Pathway consolidation of eight sets of pathway enrichment. AMR = antibody-mediated rejection, ATN = acute tubular necrosis, ACR = acute cellular rejection.

**Figure 4.**
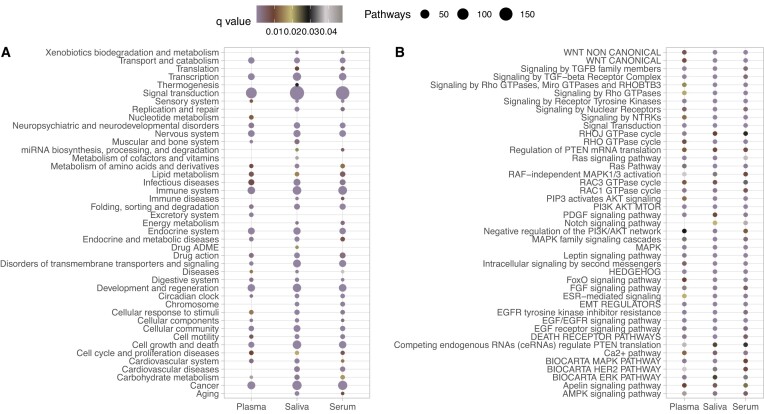
Pathways enriched in gene targets of microRNAs linked to TBI. Results are shown per biofluid. Panel **A** shows the number of pathways in each category, together with the lowest *q*-value in that category, while panel **B** shows the q-value for each pathway annotated with Signal transduction category and present in all three biofluids.

In a previous study (26) pathDIP 3 was used to perform pathway enrichment analysis in eight sets of comparisons (up- and down-regulated in glomeruli and in tubulointerstitial kidney tissues, comparing samples with antibody-mediated rejection to ones with acute tubular necrosis or acute cellular rejection), and at the time manual pathway consolidation was performed on the 1685 pathways to group them into 8 meaningful categories. We re-analysed the same genes using pathDIP 5, and we obtained 907 pathways, still a large number to get insight from. We used the default consolidation available now on the portal to recreate Figure 4A from the paper (26). In our categories, apoptosis and cell cycle are grouped in ‘Cell growth and death’, which we renamed ‘Cell cycle’ for this comparison. We also renamed our category ‘Cell motility’ to ‘ECM and cell communication’ for the same reason. As visible in Figure 3, there are some expected differences in the number of pathways for each category, but the trend observed in the original paper remains: ECM is observed only in the enrichment analysis of down-regulated genes, immune system related pathways are mainly present in the enrichment analysis of upregulated genes in glomeruli, and signal transduction is more prominent in the enrichment analysis of downregulated genes. There is a larger number of metabolic pathways in our analysis, not surprisingly as pathDIP 5 has more metabolic pathways compared to version 3 used in the original publication.

#### Pathways linked to microRNAs in traumatic brain injury

A recent study performed a metanalysis of microRNAs differentially expressed in biofluids of patients with traumatic brain injury (TBI) (27). The researchers gathered the gene targets of the microRNAs from mirDIP (28)—a database of microRNA–target interactions—and subsequently used them to perform pathway enrichment analysis using pathDIP 4. We re-run the analysis using the ‘Search miRNAs’ tab of pathDIP 5. Similarly, the majority of shared pathways are linked to signal transduction, but while the researchers used word enrichment analysis for this finding, we used the automated pathway consolidation. As visible in Figure 4A, this provides a faster way to identify which categories of pathways are shared (or exclusive) when comparing different sets. We also looked at specific signalling pathways as in the original paper and identified the same types of signalling as in the original paper, highlighting that a large reduction in the number of databases did not drastically change the message obtained in previous analyses. Figure 4B shows the *q*-value of signalling pathways present in all three biofluids, as those were the focus of the original paper discussion, and provides an unbiased and reproducible view of the pathways obtained, while in the original paper, due to the number of pathways, the researchers had to manually select the most representative candidates to highlight. We then demonstrated that using a combination of the miRNA-specific tab and the preset consolidation can provide a faster, unbiased and reproducible way to reach the same (or similar) desired results.

#### Investigation of gene coverage by source and set

PathDIP has always aimed to reduce bias and increase coverage by integrating multiple databases to expand the number of annotated genes provided and predicting pathway associations to augment the number of pathway annotations per gene as well as the number of annotated genes. This results in providing annotations also for genes that have no pathway annotation in the literature so far (what we refer to as *pathway orphans*, analogous to *interactome orphans* (29)). Using 138 genes most frequently deregulated in osteoarthritis, as described in (30), we queried pathDIP and exported the pie charts to investigate the gene coverage per source and set. As visible in Supplementary Figure 1, most of our genes have literature-curated annotation in Reactome, but still, 21% of our genes would not be annotated if we were using only Reactome, compared to 11% using all the available databases. More importantly, the 16 genes missing from the literature-curated set are clearly pathway orphans. Using pathway predictions, we can provide annotation for 4 (using only experimental PPIs) or 13 (using experimental and predicted PPIs) more genes, reaching annotation coverage for 98% of the genes of interest. Having annotations for all the genes of interest is important to ensure pathway enrichment analysis identifies pathways from the full gene set, not only a (small) fraction of genes, which leads to biased conclusions. These results also highlight one reason for irreproducible results, as results from pathway enrichment analysis often drive follow-up functional studies; and depending on which database one uses, the results could be drastically different, especially for the database-specific pathway orphans.

#### Drugs targeting specific pathways

**Figure 5.**
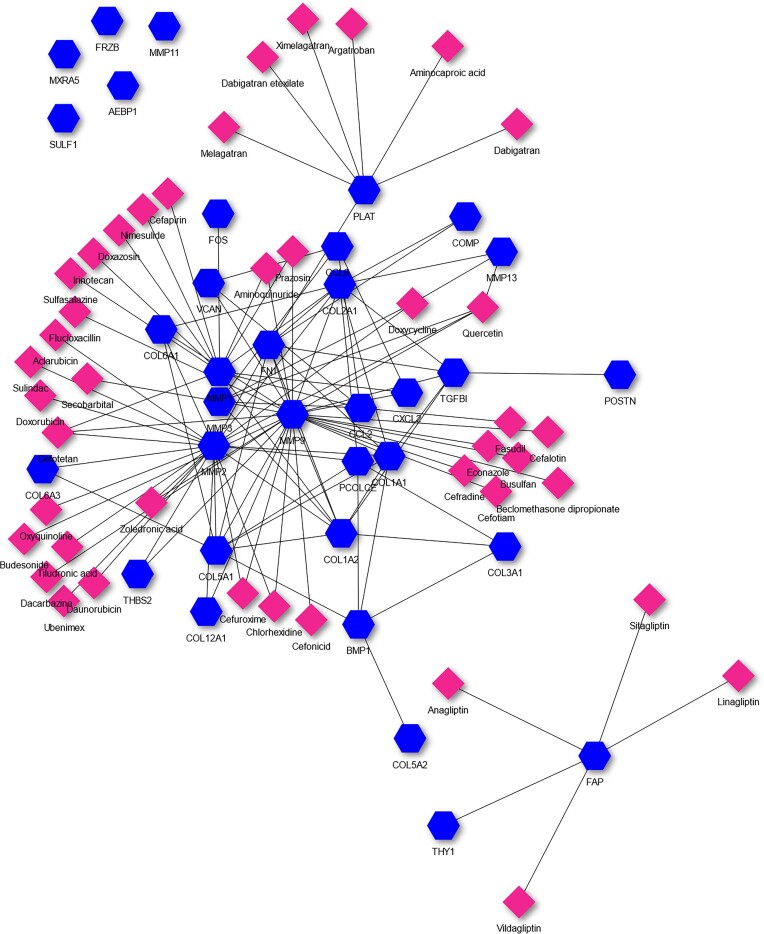
Drugs targeting the genes annotated with MATRIX REGULATION pathway. Genes are the blue hexagons while drugs are the pink diamonds.

Using the same 138 osteoarthritis-related genes as above, we performed pathway enrichment analysis using extended pathway association with experimentally detected and computationally predicted PPIs. We obtained 724 pathways, the one with the lowest p-value being ‘MATRIX REGULATION’ from ACSN2. Running Drugst.One (https://drugst.one) on this pathway and selecting to show the drugs that target the query genes in MATRIX REGULATION, we obtained the network in Figure 5. Quercetin is the drug that, in this network, targets the highest number of genes (4: MMP1, MMP3, MMP9 and MMP13). Quercetin has been shown to prevent osteoarthritis progression through the reduction of inflammation and ECM degradation, and by regulating cartilage matrix degradation and remodelling (31,32), and has been shown to be effective in improving the health of osteoarthritis patients (33).

## Discussion

PathDIP 5 addresses the shortcomings related to the number of pathways obtained by the integration of multiple databases: genome coverage, annotation biases, and statistical power.

The ability to reduce and select different kinds of pathways beforehand provides more specific results with lower adjusted p-values. No other pathway database, to our knowledge, provides the possibility to perform pathway enrichment analysis using only a subset of pathway types. It is important to note, though, that the selection should focus on reducing the noise and not increasing the bias. For example, removing disease-related pathways when studying genes linked to a specific disease reduces the noise, while selecting only metabolic pathways involved in a specific process increases the bias. A researcher needs to be aware of the difference and alert to their own biases, which should all be described in the paper to ensure reproducible science (note, results downloaded from pathDIP include those details as well).

The possibility of grouping the enriched pathways based on their type or category provides the researchers, as shown in our examples, with the ability to select subsets or showcase high-level functions performed by the genes of interest even when the number of results is quite large. This is important because it provides the ability to obtain better annotations for the genes at hand (the original aim of pathDIP) without getting overwhelmed by the number of annotations obtained. Pathway consolidation has been attempted by a few groups, and released in databases with aims different compared to pathDIP. PathMe (34) integrates only KEGG, Reactome and WikiPathways, and is focused on the exploration and visualization of integration, consensus and cross-talks among pathways. Similarly, PathCards (35) focuses on the integration and visualization of pathways from Reactome, KEGG, PharmGKB, WikiPathways, QIAGEN, HumanCyc, Pathway Interaction Database, Tocris Bioscience, GeneGO, Cell Signaling Technologies (CST), R&D Systems and Sino Biological, without providing data download or pathway enrichment analysis. ComPath (36) includes WikiPathways, KEGG and Reactome and provides the possibility to either perform enrichment analysis or visualize pathway similarity and overlap. While the data can be obtained in multiple tabs, it is harder to integrate than in pathDIP. NCATS BioPlanet (37) integrates KEGG, BioCarta, Reactome, WikiPathways, NCI-Nature, Science Signaling and NetPath, and provides pathway enrichment as well as consolidation. The portal is quite user-friendly and provides several useful annotations, but the downloaded files do not provide enrichment results. All these databases provide data only for *Homo sapiens*.

The integration with other tools, like mirDIP and Drugst.One, enables faster and more streamlined analyses. The ability to query pathDIP through API leads to integration in diverse bioinformatics workflows, providing a useful tool for bioinformaticians and computational biologists. To increase pathDIP usability and thus user base, the database now provides multiple tables and graphs, supports more immediate visualization of multiple results, and offers less computer-savvy users more options for interpretable and publication-ready results.

## Supplementary Material

gkad1027_Supplemental_Files

## Data Availability

PathDIP 5 is freely available at https://ophid.utoronto.ca/pathDIP.
