# Supplementary material for: PathDIP 5: improving coverage and making enrichment analysis more biologically meaningful
Source: Nucleic Acids Res. 2023 Nov 22;52(D1):D663–71. doi: 10.1093/nar/gkad1027 (PMC10767947; doi:10.1093/nar/gkad1027)
Supplement: gkad1027_Supplemental_Files [file gkad1027_supplemental_files.zip › Supplement table 2.pdf]

Type	Category
Cellular processes and organization	Cell growth and death
Cellular processes and organization	Cellular community
Cellular processes and organization	Cellular components
Cellular processes and organization	Cellular response to stimuli
Cellular processes and organization	Transport and catabolism
Diseases	Cancer
Diseases	Cardiovascular diseases
Diseases	Cell cycle and proliferation diseases
Diseases	Diseases
Diseases	Disorders of transmembrane transporters and signaling
Diseases	Endocrine and metabolic diseases
Diseases	Hemostasis diseases
Diseases	Immune diseases
Diseases	Infectious diseases
Diseases	Neuropsychiatric and neurodevelopmental disorders
Drugs and vitamins	Drug action
Drugs and vitamins	Drug action and ADME
Drugs and vitamins	Drug ADME
Drugs and vitamins	Vitamin action
Environmental information processing	Membrane transport
Environmental information processing	Signal transduction
Genetic information processing	Chromosome
Genetic information processing	Folding, sorting and degradation
Genetic information processing	miRNA biosynthesis, processing, and degradation
Genetic information processing	Replication and repair
Genetic information processing	Transcription
Genetic information processing	Translation
Metabolism	Biosynthesis of secondary metabolites
Metabolism	Carbohydrate metabolism
Metabolism	Energy metabolism
Metabolism	Lipid metabolism
Metabolism	Metabolism
Metabolism	Metabolism of amino acids and derivatives
Metabolism	Metabolism of cofactors and vitamins
Metabolism	Metabolism of terpenoids and polyketides
Metabolism	Microbial metabolism in diverse environments
Metabolism	Nucleotide metabolism
Metabolism	Xenobiotics biodegradation and metabolism
Organismal systems	Aging
Organismal systems	Cardiovascular system
Organismal systems	Cell motility
Organismal systems	Cell system
Organismal systems	Circadian clock
Organismal systems	Development and regeneration
Organismal systems	Digestive system
Organismal systems	Endocrine system

Organismal systems	Excretory system
Organismal systems	Immune system
Organismal systems	Muscular and bone system
Organismal systems	Nervous system
Organismal systems	Reproductive system
Organismal systems	Sensory system
Organismal systems	Thermogenesis
