# Supplementary material for: PathDIP 5: improving coverage and making enrichment analysis more biologically meaningful
Source: Nucleic Acids Res. 2023 Nov 22;52(D1):D663–71. doi: 10.1093/nar/gkad1027 (PMC10767947; doi:10.1093/nar/gkad1027)
Supplement: gkad1027_Supplemental_Files [file gkad1027_supplemental_files.zip › Supplementary Figure 1.pdf]

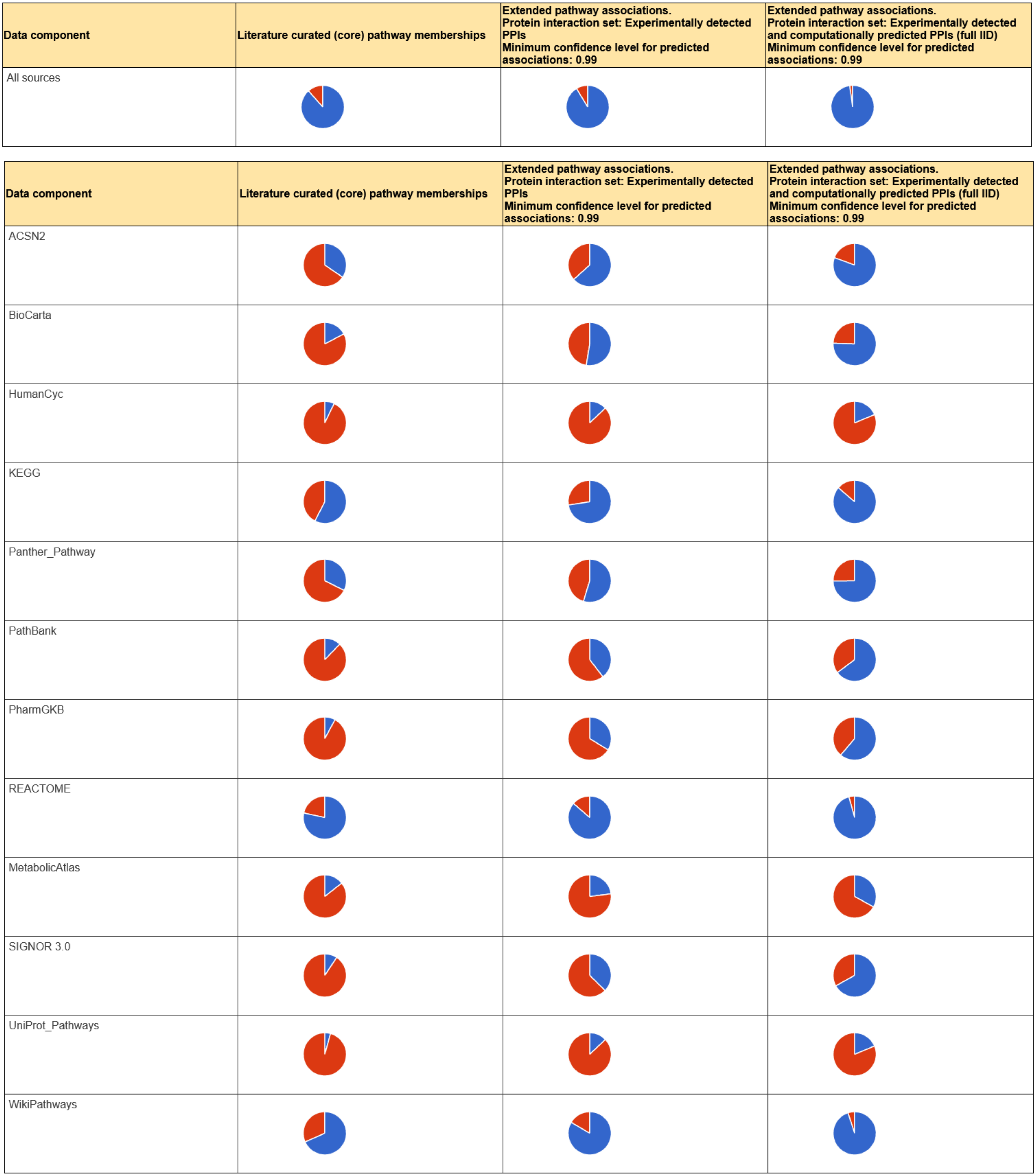

Supplementary Figure 1. Number of genes annotated with any pathway in any of the sources (top row), or each source (bottom rows). Blue indicates annotated genes, red indicates genes absent in that source or set.
