# Supplementary material for: PathDIP 5: improving coverage and making enrichment analysis more biologically meaningful
Source: Nucleic Acids Res. 2023 Nov 22;52(D1):D663–71. doi: 10.1093/nar/gkad1027 (PMC10767947; doi:10.1093/nar/gkad1027)
Supplement: gkad1027_Supplemental_Files [file gkad1027_supplemental_files.zip › Supplementary Table 1new.pdf]

Sources of experimentally detected PPIs

Source	Version	Citation
BCI		(1)
BioGRID	4.3.196	(2)
DIP		(3)
HPRD	9	(4)
InnateDB	5.4	(5)
IntAct	4.2.16	(6)
MatrixDB		(7)
MINT		(8)

Sources of computationally predicted PPIs

Source	Citation	Scores of included PPIs
BCI	(1)	probability > 0.5
Elefsinioti et al.	(9)	probability > 0.7
Kotlyar et al.	(10)	false discovery rate < 0.6
Rhodes et al.	(11)	likelihood ratio ≥ 381
Zhang et al.	(12)	likelihood ratio ≥ 600

Orthology-based PPIs

IID predicts orthologous PPIs by mapping experimentally detected PPIs to orthologous PPIs in other species, using 1:1 orthologs from Ensembl (13) release 103.

References

1. Lefebvre,C., Lim,W.K., Basso,K., Dalla Favera,R. and Califano,A. (2007) A context-specific network of protein-DNA and protein-protein interactions reveals new regulatory motifs in human B cells. In *Lecture Notes in Computer Science (including subseries Lecture Notes in Artificial Intelligence and Lecture Notes in Bioinformatics)*.Vol. 4532 LNBI, pp. 42–56.
2. Oughtred,R., Rust,J., Chang,C., Breitkreutz,B.J., Stark,C., Willems,A., Boucher,L., Leung,G., Kolas,N., Zhang,F., *et al.* (2021) The BioGRID database: A comprehensive biomedical resource of curated protein, genetic, and chemical interactions. *Protein Science*, **30**, 187–200.
3. Salwinski,L., Miller,C.S., Smith,A.J., Pettit,F.K., Bowie,J.U. and Eisenberg,D. (2004) The Database of Interacting Proteins: 2004 update. *Nucleic Acids Res*, **32**, D449-51.

4. Keshava Prasad,T.S., Goel,R., Kandasamy,K., Keerthikumar,S., Kumar,S., Mathivanan,S., Telikicherla,D., Raju,R., Shafreen,B., Venugopal,A., *et al.* (2009) Human Protein Reference Database--2009 update. *Nucleic Acids Res*, **37**, D767-72.
5. Breuer,K., Foroushani,A.K., Laird,M.R., Chen,C., Sribnaia,A., Lo,R., Winsor,G.L., Hancock,R.E.W., Brinkman,F.S.L. and Lynn,D.J. (2013) InnateDB: systems biology of innate immunity and beyond--recent updates and continuing curation. *Nucleic Acids Res*, **41**, D1228-33.
6. Orchard,S., Ammari,M., Aranda,B., Breuza,L., Briganti,L., Broackes-Carter,F., Campbell,N.H., Chavali,G., Chen,C., del-Toro,N., *et al.* (2014) The MIntAct project--IntAct as a common curation platform for 11 molecular interaction databases. *Nucleic Acids Res*, **42**, D358-63.
7. Clerc,O., Deniaud,M., Vallet,S.D., Naba,A., Rivet,A., Perez,S., Thierry-Mieg,N. and Ricard-Blum,S. (2019) MatrixDB: integration of new data with a focus on glycosaminoglycan interactions. *Nucleic Acids Res*, **47**, D376–D381.
8. Licata,L., Briganti,L., Peluso,D., Perfetto,L., Iannuccelli,M., Galeota,E., Sacco,F., Palma,A., Nardozza,A.P., Santonico,E., *et al.* (2012) MINT, the molecular interaction database: 2012 Update. *Nucleic Acids Res*, **40**.
9. Elefsinioti,A., Saraç,Ö.S., Hegele,A., Plake,C., Hubner,N.C., Poser,I., Sarov,M., Hyman,A., Mann,M., Schroeder,M., *et al.* (2011) Large-scale de novo prediction of physical protein-protein association. *Mol Cell Proteomics*, **10**, M111.010629.
10. Kotlyar,M., Pastrello,C., Pivetta,F., Lo Sardo,A., Cumbaa,C., Li,H., Naranian,T., Niu,Y., Ding,Z., Vafaee,F., *et al.* (2015) In silico prediction of physical protein interactions and characterization of interactome orphans. *Nat Methods*, **12**, 79–84.
11. Rhodes,D.R., Tomlins,S.A., Varambally,S., Mahavisno,V., Barrette,T., Kalyana-Sundaram,S., Ghosh,D., Pandey,A. and Chinnaiyan,A.M. (2005) Probabilistic model of the human protein-protein interaction network. *Nat Biotechnol*, **23**, 951–9.
12. Zhang,Q.C., Petrey,D., Deng,L., Qiang,L., Shi,Y., Thu,C.A., Bisikirska,B., Lefebvre,C., Accili,D., Hunter,T., *et al.* (2012) Structure-based prediction of protein-protein interactions on a genome-wide scale. *Nature*, **490**, 556–60.
13. Howe,K.L., Achuthan,P., Allen,J., Allen,J., Alvarez-Jarreta,J., Amode,M.R., Armean,I.M., Azov,A.G., Bennett,R., Bhai,J., *et al.* (2021) Ensembl 2021. *Nucleic Acids Res*, **49**, D884–D891.
