# Supplementary material for: PathDIP 5: improving coverage and making enrichment analysis more biologically meaningful
Source: Nucleic Acids Res. 2023 Nov 22;52(D1):D663–71. doi: 10.1093/nar/gkad1027 (PMC10767947; doi:10.1093/nar/gkad1027)
Supplement: gkad1027_Supplemental_Files [file gkad1027_supplemental_files.zip › Table S2 legends.docx]

Table S2 is: Mapping between types and categories for
PathDIP 5 consolidation
